# Spatial transcriptomic profiling identifies lacrimal-gland-epithelial cell-driven mechanisms underlying autoimmunity in Sjögren’s disease

**DOI:** 10.3389/fimmu.2026.1759347

**Published:** 2026-03-05

**Authors:** Shivali Gupta, Athanasios Ploumakis, Nikolaos Kalavros, Sharmila Masli

**Affiliations:** 1 Department of Ophthalmology, Boston University Chobanian & Avedisian School of Medicine, Boston, MA, United States; 2 Spatial Technologies Unit, Beth Israel Deaconess Medical Center, Harvard Medical School Initiative for RNA Medicine, Boston, MA, United States

**Keywords:** acinar epithelial cells, antigen presenting cells, autoimmunity, duct epithelial cells, lacrimal gland, Sjögren’s disease, spatial transcriptomics

## Abstract

**Introduction:**

Sjögren’s disease (SjD) is the second most prevalent rheumatic disease and is characterized by autoimmune pathology targeting the tear-producing lacrimal glands, leading to chronic ocular surface disease. Despite important advances, lacrimal gland pathology in SjD remains incompletely understood, limiting both diagnosis and treatment.

**Methods:**

In this exploratory study, we used spatial transcriptomics to profile lacrimal glands from wild-type (C57Bl/6) mice and thrombospondin-1-deficient (TSP-1^-^/^-^) mice, a spontaneous model of SjD, to identify molecular signatures associated with the functional loss of major epithelial cell subtypes—acinar, ductal, and myoepithelial cells.

**Results:**

Our analyses revealed gene expression patterns consistent with endoplasmic reticulum stress in acinar cells, mitochondrial dysfunction in ductal epithelial cells, secretory dysfunction in both acinar and ductal epithelial cells, and contractile impairment with profibrotic remodeling in myoepithelial cells in SjD lacrimal glands, highlighting potential early mechanisms and markers of glandular damage. Furthermore, in acinar epithelial cells, a significantly reduced expression of Pigr, which encodes the polymeric immunoglobulin receptor required for the transcytotic delivery of protective secretory IgA into tear fluid, correlated with reduced tear secretory IgA levels in SjD mice, consistent with their observed ocular surface disease.

**Discussion:**

This finding supports the potential use of tear sIgA as a quantifiable biomarker of glandular dysfunction. By integrating spatial and cellular information, we uncovered a previously unrecognized spatial relationship between ductal epithelial cells and antigen-presenting cells in the lacrimal gland and identified a potential role for ductal epithelial cells as active drivers of inflammation by providing molecular and cellular cues that support periductal infiltrates rich in B cells and T follicular helper cells that form germinal centers and promote local autoantibody production. These findings together generate testable mechanistic hypotheses for each epithelial subtype and propose a framework for the therapeutic targeting of epithelial cells and multicellular interactions that underlie autoimmune lacrimal gland pathology in SjD.

## 1 Introduction

Sjögren’s disease (SjD) is the second most prevalent autoimmune rheumatic disease characterized primarily by chronic inflammation and dysfunction of exocrine glands like lacrimal and salivary glands. Additionally, extra-glandular manifestations involving joints, skin, lungs, and nervous system due to systemic autoimmunity are observed in up to 75% of patients (1). Ocular dryness or dry eye disease (DED) is one of the most common clinical manifestations of SjD resulting from lacrimal gland (LG) dysfunction and damage (2, 3). Due to insidious onset, morbidity in SjD patients increases progressively with age, leading to debilitating symptoms and increased risk of mortality with extra-glandular manifestations, including lymphoma (2, 4). Current management of SjD involves treatments for symptomatic relief, while there is no approved disease-modifying anti-rheumatic drug (DMARD) available (5). Despite the progress made in elaborating immunopathogenic mechanisms underlying SjD, the molecular basis of multicellular interactions in the lacrimal gland spatial architecture and tissue microenvironment that contribute to the autoimmune disease and its progression are still not fully understood, limiting therapeutic innovation in SjD.

Structurally, the lacrimal gland (LG) is composed of multiple lobules that contain secretory units, acini, and an interlobular and intralobular duct system. Acinar epithelial cells are primary secretory cells that produce protein-rich lacrimal fluid, while the duct epithelial cells modify this primary fluid via absorption or secretion of ions, thereby finetuning tear composition before delivery to the ocular surface. Contractile myoepithelial cells (MECs) wrap around both acini and ducts to facilitate the expulsion of tear fluid from the gland (6). In addition to these epithelial cells, LGs also harbor innate and adaptive immune cells involved in immune defense and surveillance within the gland. Immune cells like plasma cells produce secretory IgA (sIgA) (6). The combined action of sIgA, antimicrobial factors, and other proteins secreted by acinar epithelial cells creates tear fluid that is both protective and essential to maintain the health and integrity of the ocular surface. Lacrimal gland dysfunction in SjD is known to compromise aqueous tear production and quality. While glandular damage is largely attributed to local inflammatory immune responses it is not known if and how lacrimal gland epithelial cells contribute to modulation of local immune responses and how their functional alterations collectively contribute to the pathogenesis of SjD.

In our study, we used a TSP-1-deficient mouse model of SjD that spontaneously develops the disease with characteristic LG inflammatory infiltrates, functional loss along with ocular surface dryness, and SjD-related autoantibodies as observed in human patients (7, 8). As a matricellular glycoprotein, TSP-1 regulates cell–matrix interactions, activation of latent TGFβ, immune homeostasis, and tissue remodeling. Loss of TSP-1 contributes to ocular disease by disrupting the regulation of immune, angiogenic as well as lymphangiogenic responses as reviewed elsewhere (9) and predisposes LG to glandular dysfunction characteristic of SjD. In a study designed to dissect non-immune events in a NOD mouse model of SjD, a microarray analysis of LG tissue from NOD/scid mice was performed (10). It is noteworthy that a search of their GEO Profiles database (11) revealed a reduced expression of TSP-1 in LGs of NOD/scid mice (GEO accession GDS2177 (10)), suggesting the relevance of reduced intrinsic TSP-1 expression with disease development. Similarly, in a recent transcriptomic analysis of minor salivary glands of SjD patients, a significantly reduced expression of TSP-1 was detectable [GEO accession GSE157159 (12)]. This result is consistent with the reduced TSP-1 expression detectable in the salivary glands of patients with advanced SjD as compared to healthy controls [GEO accession GDS3940 (13)]. Collectively, these studies further validate the clinical relevance of TSP-1 deficiency in SjD pathogenesis.

Recently, several studies applied single-cell RNA-seq (scRNA-seq) to help identify diverse epithelial and immune cell populations in LGs of healthy and SjD mouse models (14–16).

These studies highlight complex heterogeneity among glandular cell populations and immune cells, suggesting the need for further analysis to clarify cell-type-specific contributions and molecular changes. In this exploratory study, we integrated cell-type signatures and expression programs with whole transcriptome digital spatial profiles of matched specimens of LG tissues from healthy and TSP-1-deficient SjD mouse models. This approach allowed us to identify spatially resolved expression patterns and glandular cell–cell interactions in an assumption-free manner. While immune-mediated mechanisms have been widely studied in autoimmune diseases, our study specifically highlights the underexplored contribution of lacrimal gland epithelial cells to the autoimmune pathogenesis of Sjögren’s disease. By integrating spatial transcriptomic signatures with known clinical manifestations—such as altered tear film composition, increased osmolarity, and the development of autoantibody responses—we aimed to establish a system-level framework linking molecular changes with functional outcomes. We conducted orthogonal validation of selected transcriptomic findings at the protein level, which supports the overall biological relevance of our observations. We identified several distinct molecular alterations in glandular epithelial cells that correlate with the pathogenesis. Furthermore, we discovered spatially defined interactions between glandular epithelial cells and immune cells that can support the development of local adaptive immune response. These cellular mechanisms are expected to not only serve as a foundation for more focused follow-up studies targeting specific pathways in epithelial subtypes uncovered in this work but also identify potential targets for therapeutic innovation in SjD.

## 2 Materials and methods

### 2.1 Animals

Twelve-week-old male C57BL/6 and B6.129S2-Thbs1<tmlHyn>/J (TSP1^-/-^) mice were purchased from Jackson Laboratories (Bar Harbor, ME, USA). The Institutional Animal Care and Use Committee (IACUC) at Boston University School of Medicine, Boston, approved the animal studies described in this manuscript (protocol number IPROTO202200000063) in accordance with the National Institutes of Health (NIH) guide for the care and use of laboratory animals. All animal experiments were conducted in accordance with the Association for Research in Vision and Ophthalmology (ARVO) statement for the Use of Animals in Ophthalmic and Vision Research.

Both male and female TSP-1^-/-^mice develop SjD and associated LG pathology (7, 17). Systematic and extensive characterization of clinical parameters related to the progressive development of the ophthalmic disease, together with temporal immunopathogenic changes in T and B cell compartments, has been reported in male TSP-1^-/-^mice (18). Therefore, we used male mice in this study to leverage these established datasets and to enable direct correlations at the cellular and molecular levels with clinical parameters that are common to SjD patients and TSP-1^-^/^-^ mice. This approach provides a well-validated framework to interpret our mechanistic findings, while future work can address potential sex-specific differences more directly.

### 2.2 Digital spatial profiling

In this exploratory study, serial sections of formalin-fixed paraffin-embedded (FFPE) LG tissues harvested from 14-week-old WT (*n*= 3) and TSP^-/-^(*n*= 4) mice were cut (5 µm thick) and transferred to slides.

One set of slides was stained for H&E, and another set of slides with consecutive sections were submitted for spatial profiling to the Beth Israel Deaconess Medical Center (BIDMC) Spatial Technologies Unit (STU), MA, USA. The GeoMX Digital Spatial Profiler (DSP) platform was used to generate spatially resolved transcriptomic data, specifically the Whole Transcriptome Atlas (WTA) mouse RNA probe set for Illumina Systems (GMX-RNA-NGS-MsWTA-4) assay. This platform allows an unbiased view of the whole mouse transcriptome as the WTA assay includes RNA probe set for 21,000+ transcripts for mouse-protein-coding genes plus negative controls designed for Illumina NGS readout with the Seq Code library prep. To visualize tissue structural components and microenvironment, a set of fluorophore-conjugated morphology markers were used. These included Alexa Fluor 532 (green)-conjugated anti-pan-CK (AE1+AE3, Novus, cat. no. NBP2-33200), Alexa Fluor 594 (yellow)-conjugated anti-CD45 (EM-05, Novus, cat. no. NBP1-44763AF594), and Alexa Fluor 647 (red)-conjugated anti-smooth muscle actin (SP171, Abcam, Cat.# ab267537) and nuclear stain SYTO13 (blue). These markers were used to delineate the nuclear, epithelial (acinar and duct), immune, and myoepithelial compartments. Immunofluorescence imaging, region of interest (ROI) selection, segmentation into marker-specific areas of interest (AOI), and spatially indexed barcode cleavage and collection were performed on a GeoMx Digital Spatial Profiling instrument (NanoString). Selected ROIs were exposed to ultraviolet (UV) light to cleave DNA tags in a region-specific manner. The released indexing oligos were then collected using microcapillary aspiration and dispensed into a microplate as per segmentation of each ROI.

Considering the small size of mouse LG, four consecutive sections per tissue (total 16 of TSP-1^-/-^and 12 of WT) were assessed to enhance spatial coverage and technical robustness. Approximately, six to 10 ROIs and 11–14 AOIs per tissue sample (total 34 ROIs and 56 AOIs for TSP-1^-/-^and 21 ROIs and 34 AOIs for WT) were collected. All ROIs were within the defined area limit set in GeoMx platform (0.52 mm^2^). Library preparation was performed as per the manufacturer’s instructions, which included PCR amplification to add Illumina adapter sequences and unique dual sample indices. Sequencing was performed to a minimum of 350M PE reads by pooling all 91 uniquely indexed WTA AOIs and sequencing on Illumina NovaSeq 6000 S4 flowcell in a 2 × 150-bp PE, dual index, and 8bp i5 and i7 index length configuration.

### 2.3 DSP data preprocessing and analysis

Processing of RNA sequencing files (FASTQ files) from Illumina sequencer according to parameters defined in the configuration file generated from the GeoMX DSP run was performed using the GeoMx NGS Pipeline v2.0 (Bruker Spatial Biology, formerly NanoString Technologies, Inc., WA, USA) to produce digital count conversion (.dcc) files. Statistical analyses treat genotype (WT vs TSP-1^-/-^) as a fixed factor, with AOIs/segments nested within animals, so that animal-level independence is preserved. To minimize selection bias and ensure data quality, all presented analyses were restricted to specific AOIs that met stringent QC thresholds per NanoString guidelines (>1,000 detection counts, >80% reads aligned, >50% sequencing saturation). Counts were loaded into R Statistical Software (v4.3.2) (19), whereupon they were normalized and evaluated for differential expression using DESeq2 (20), filtering out all unannotated genes (Rik and Gm), applying a median count threshold of five per group for independent expression filtering and utilizing Storey’s *q*-values for multiple hypothesis correction (21). Subsequently, ClusterProfiler (22) was used to carry out over-representation (ORA) and gene set enrichment (GEO) analysis for pathways. Gene visualizations were done using variance-stabilized transform normalized counts or log-counts. Cell type proportions in ROIs were estimated using Multi-subject Single Cell (MuSiC) deconvolution analysis toolkit (23) with a publicly available dataset GSE132420 as a reference (15). For receptor–ligand correlation across ROIs, known receptor–ligand pairs were obtained from CellChatDB (24), and potential receptor–ligand pairs were quantified using the Spearman rank correlation between paired segments within each ROI. To compare the magnitude of correlations between WT and TSP-1-/- tissues, differential correlation analysis of cell types across conditions was performed and cell type rich segments were paired per ROI per condition. The cocor R package (25) was then used for a statistical comparison of correlations. Plots were generated using ggplot2 (26), ggpubr (27), and pheatmap (28).

### 2.4 Immunofluorescence staining

To validate the expression of the selected genes of interest, 5-µm-thick FFPE sections of LGs were immunostained using specific antibodies. These slides with sections were baked at 60°C overnight, deparaffinized with xylene (10 min; twice), rehydrated, and subjected to heat-induced antigen retrieval (HIER) in a citrate-based buffer, pH 6 (Vector Laboratories, CA, USA) for 25 min in a microwave. After antigen retrieval, the slides were photobleached, and sections were blocked with 10% normal goat or donkey serum for 1 h, followed by 2% bovine serum albumin (Sigma Aldrich, St Louis, USA) in 0.3% Triton-X100 in PBS for 30 min at room temperature (RT). The sections were incubated overnight at 4°C with 1:50 dilution in PBS-BSA of the primary antibodies listed in **Supplementary Table 1**, followed by three washes with PBS-0.05%Tween20 (2 min each). Tissues were then incubated for 1.5 h at RT in the dark with the fluorescence-conjugated secondary antibody (1:500-1:1,000) listed in **Supplementary Table 1**, washed three times with PBS-0.05%Tween20 (5 min each), counterstained with DAPI, and mounted using ProLong Gold Antifade Mountant (Invitrogen, USA). For dual-immuno-staining, sequential staining was performed (Novus Biologicals Ref). For immunostaining of spleen sections, similar steps were followed, except in the case of CD4 staining zinc fixative (BD Biosciences, CA, USA) that was used without antigen retrieval steps.

In case of primary cell cultures, cells grown on coverslips were fixed in ice-cold methanol for intracellular targets like cytokeratin and smooth muscle actin and with 2% PFA for cell surface target like CFTR. Fixed cells were incubated at RT for 15 min with blocking buffer containing 2% BSA/1% Triton X-100 in PBS for intracellular targets and for 1 h with 2% BSA in PBS for cell surface target. After three washes with PBS, the cells were incubated with primary antibodies at RT for 3 h, followed by 1 h with fluorescence-conjugated secondary antibody. After final washes with PBS, the cells were mounted in mounting medium containing nuclear stain DAPI for microscopic examination.

Images were acquired using a fluorescence microscope (Nikon Eclipse E800, Nikon, Japan) equipped with a MicroPublisher 6 camera and further processed using Fiji ImageJ software (National Institutes of Health, USA). Fluorescence intensity was determined as corrected total cell fluorescence (CTCF) calculated as [integrated density of stained tissue – (area of stained tissue × mean fluorescence of background reading)].

### 2.5 Primary lacrimal gland duct epithelial cell culture

Keratinocyte basal medium (KBM) and RPMI-1640 (Lonza) supplemented with 10% heat inactivated fetal calf serum (Thermo Fisher Scientific, Waltham, MA), 1 mM sodium pyruvate, 10 mM HEPES, 100 mg/mL penicillin–streptomycin, and 0.1 mM NEAA (Sigma Aldrich, St. Louis, MO) were used in 1:1 proportion for cell cultures. The lacrimal gland tissues were minced into small pieces and were anchored onto scored 48-well culture plates. Three pieces of tissue were anchored per culture well with approximately 75 mL medium to cover the bottom of the well. The culture plates were incubated under routine culture conditions of 5% CO_2_at 37°C. The medium was replaced every 2 to 3 days, and after tissue pieces adhered to the plastic, the medium volume was increased to 100 mL. Cell growth was monitored routinely until it reached >75% confluence in 10–14 days, after which the explants were removed. The cells were used either for immunostaining or culture supernatants were collected for ELISA. In some experiments, the cells were treated for 24 h with recombinant mouse IFN-g (10 ng/mL, R&D Systems, Minneapolis, MN, USA).

### 2.6 RT-PCR

Total RNA isolated from untreated or IFN-γ-treated epithelial cell cultures using TRIzol reagent (Life Technologies, Carlsbad, CA, USA) was used to synthesize cDNA using SuperScript VILO cDNA synthesis kit (Thermofisher Scientific, Waltham, USA) according to the manufacturer’s instructions. For amplification of MHC class II-specific gene transcripts, primer sets F-5′- AGG GCA TTT CGT GTA CCA GTT and R-5′-GTA CTC CCG GTT GTA GAT GTA were used along with primers for the reference gene glyceraldehyde-3-phosphate dehydrogenase primers (F-5′- CGAGAATGGGAAGCTTGTCA-3′ and R-5′-AGACACCAGTAGACTCCACGACAT-3′). Real-time PCR assay was performed using SYBR Green PCR Master Mix (Thermofisher Scientific, Waltham, USA) with amplification reactions set up in quadruplicates and thermal profile as follows: 95°C for 3 min, 40 cycles at 95°C for 10 s, 54.5°C for 10 s, and 72°C for 30 s. The specificity of the amplification reaction was verified by performing melting curve analysis. The threshold cycle values were used to determine the quantification of gene expression relative to the reference gene GAPDH.

### 2.7 ELISA

Culture supernatants collected from primary cultures of WT and TSP-1^-/-^LG epithelial cells were analyzed for IL-6 content using ELISA (eBioscience, CA, USA) according to the manufacturer’s instructions. Pilocarpine-induced tear samples collected from 24-week-old mice were analyzed for their sIgA content using ELISA (Novus Biologicals, CO, USA).

### 2.8 Statistical analysis

Student’s unpaired *t*-test was used to determine significant differences between mean values of experimental and control groups, and *p*< 0.05 was considered significant. Statistical analysis was performed using GraphPad Prism 10 software.

## 3 Results

### 3.1 Mapping cell types and transcriptional programs to lacrimal gland architecture

**Figure 1:**
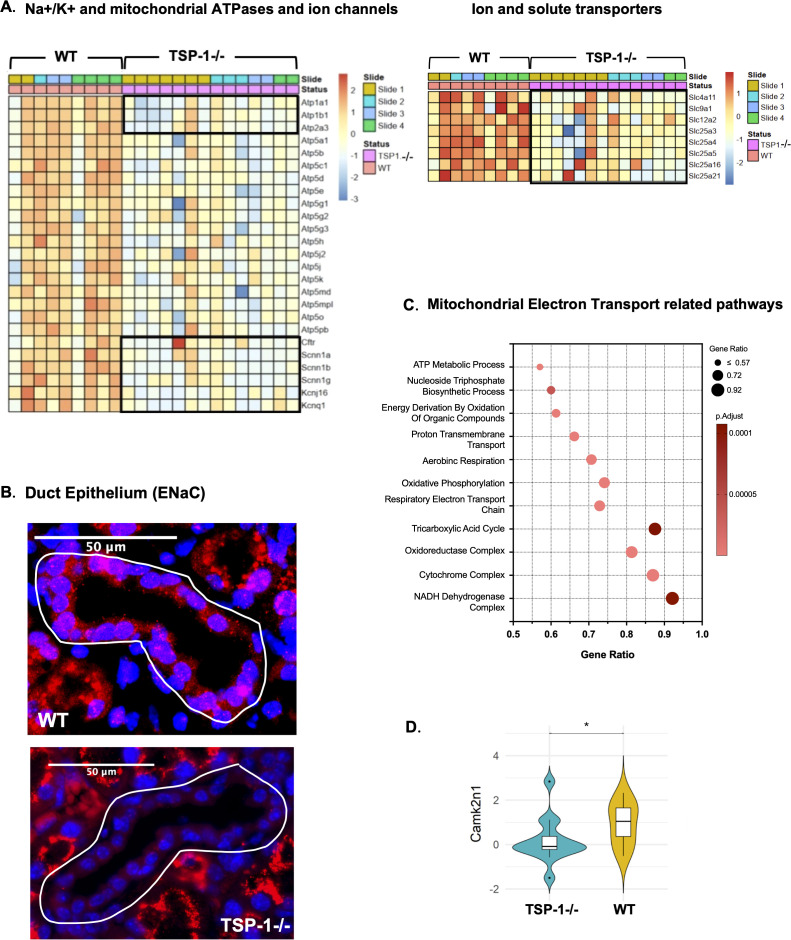
Digital spatial profiling (DSP) and whole transcriptome analysis (WTA) of mouse lacrimal gland tissues. **(A)** Representative hematoxylin and eosin (H&E)-stained FFPE section (5 µm thick) of LG from TSP-1^-/-^mice showing representative areas selected to identify the region of interest (ROI) that contained acini with surrounding myoepithelial cells (upper left), ducts (lower left), and periductal and perivascular inflammatory infiltrates (upper and lower right). **(B)** Representative immunofluorescence images (GeoMx DSP) of a consecutive section from the same FFPE block stained for PanCK (green), CD45 (yellow), α-SMA (red), and nuclear stain SYTO13 (blue) and ROIs. Segmentation was used to identify areas of interest (AOIs) to enrich for acinar, myoepithelial, duct, and immune cells based on staining for indicated markers and morphology. **(C)** UMAP plot showing cell clusters identified in ROIs from WT and TSP-1^-/-^LGs. **(D)** UMAP plots for indicated transcripts that identified specifically one or two clusters. **(E)** Heatmap showing major immune cell populations (T cells, B cells, plasma cells, and monocytes) detected in a total of 90 AOIs (34 WT and 56 TSP-1^-/-^ LGs). The color scale indicates relative expression levels.

To determine the transcriptional programs of the structural components of LG in the context of their spatial organization, we performed digital spatial profiling with the NanoString GeoMx mouse whole transcriptome atlas (WTA; 21,000+ genes) in this exploratory study. We used formalin-fixed paraffin-embedded (FFPE) sections of LG tissues from TSP-1^-/-^and WT mice. Characteristic periductal and perivascular mononuclear infiltrates were detectable in H&E-stained sections of TSP-1^-/-^but not normal WT LGs (**Figure 1A**). *In situ*hybridization was performed on these tissues using UV-photocleavable barcode-conjugated RNA probes. From selected regions of interest (ROI), mRNA counts were captured and profiled. These ROIs included acini with surrounding myoepithelial cells (MECs) or ducts and surrounding mononuclear infiltrates (**Figure 1A**). Overall, areas of ROIs in TSP-1^-/-^and WT tissues were comparable (mean ± SEM, 0.046 ± 0.007 vs. 0.031 ± 0.005 mm^2^, respectively, *p*> 0.05). Within each ROI, using four-color immunofluorescence and morphological appearances of cell types, custom areas of illumination (AOIs) were identified for each cell-type segment (**Figure 1B**). Barcodes cleaved and collected from each AOI or segment were quantified by sequencing. To address the previously reported heterogeneity of LG tissue, we used higher sequencing depth and multiple replicates of tissue samples to reliably identify differentially expressed genes (DEGs) and help resolve signals from relatively less abundant cell types (29, 30). We analyzed a total of 28 LG tissue samples (**Supplementary Figure 1**) that included 16 sections from TSP-1^-/-^(*n*= 4) and 12 from WT (*n*= 3) mice. For each LG tissue, four sections from different regions were collected to increase spatial coverage and technical robustness while still preserving animal-level independence. Over 27,500 transcripts obtained from 32,465 cells were sequenced, and NGS data was deconvolved using scRNA-seq cell-type signatures from publicly available data set (GSE132420) as a reference. A total of 12 principal cell clusters were detected (**Figure 1C**), and their identity was validated using published genes associated with each cell type (**Figure 1D**). When mapped onto ROIs, 87% of the genes of cell type programs were detected above background. Transcriptional programs in AOIs for acinar, duct, and myoepithelial cells were more variable between groups than within each group (**Supplementary Figure 2**). Among epithelial cells, we detected most DEGs in acinar epithelial cells, followed by duct and myoepithelial cells (**Table 1**). In acinar epithelial cells, most DEGs were upregulated, while in duct epithelial cells they were predominantly downregulated, and in MECs equal proportion of DEGs were up- and downregulated in TSP-1^-/-^LGs. Furthermore, comparisons of the major immune cell populations (T cells comprising T and NK cells, B cells, plasma cells, and monocytes comprising macrophages and dendritic cells) in WT (34 AOIs) and TSP-1^-/-^(56 AOIs) revealed more AOIs containing B cells, plasma cells as well as T cells in TSP-1^-/-^glands (**Figure 1E**).

**Table 1:** Gene expression pattern in WT and TSP-1^-/-^lacrimal gland epithelial cells.

Epithelial cell type	Numbers of Differentially Expressed Genes (Adj p < 0.005)			
	Total	Significantly altered	Upregulated	Downregulated
Acinar	19300	2052 (11%)	1182 (58%)	870 (42%)
Duct	3259	445 (14%)	140 (32%)	305 (68%)
Myoepithelial	4965	296 (6%)	148 (50%)	148 (50%)

### 3.2 ER stress pathways in TSP-1-deficient acinar epithelial cells disrupt their secretory function and promote autoimmune pathology

**Figure 2:**
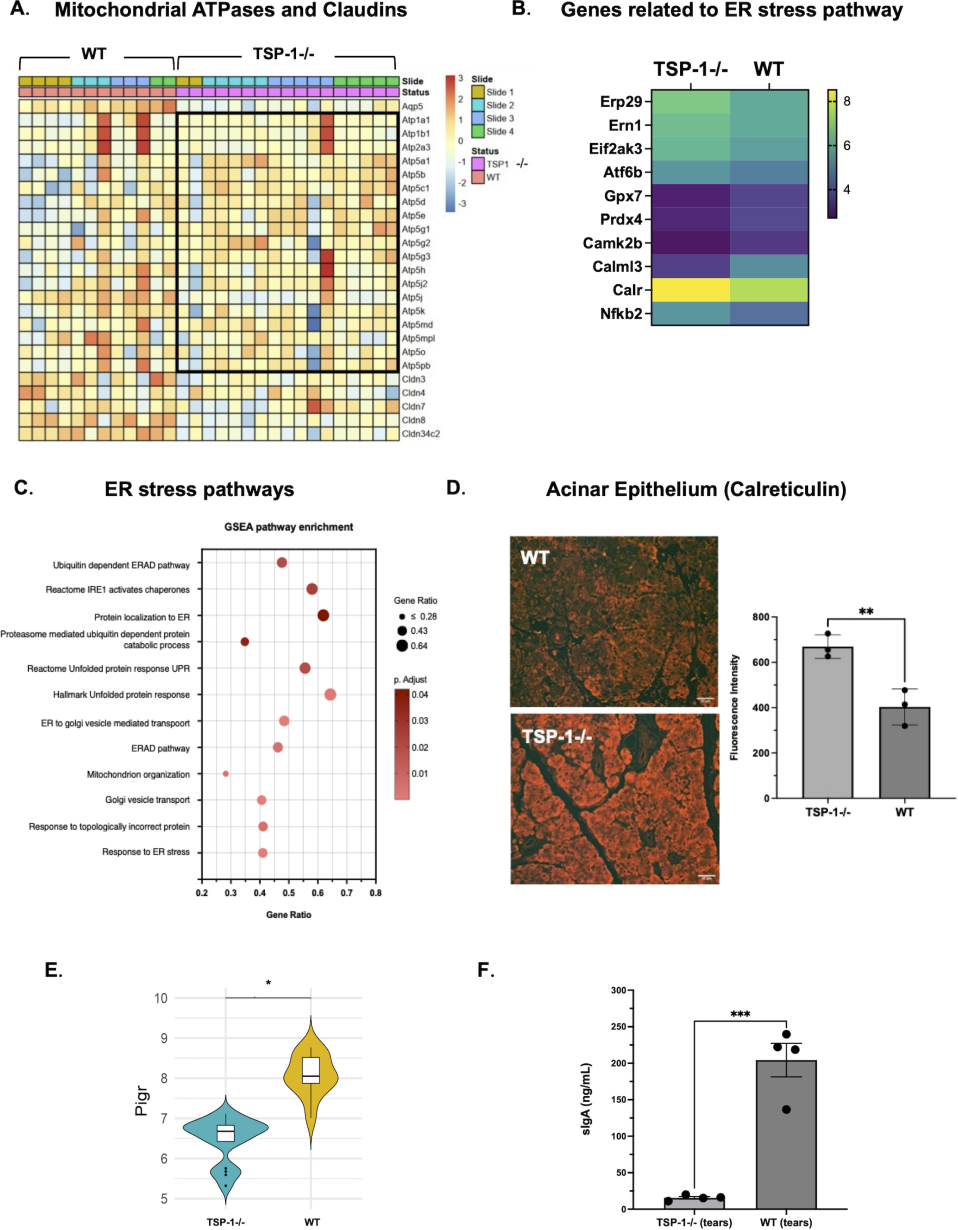
Endoplasmic reticulum (ER) stress pathways in TSP-1-deficient acinar epithelial cells and associated secretory dysfunction. **(A)** Heatmap of the overall gene expression pattern of ATPases and claudins in acinar epithelial cell segments (12 WT and 16 TSP-1^-/-^). The black box highlights mitochondrial ATPases. **(B)** Heatmap illustrating the expression of DEGs associated with ER stress response pathways (adj *p*< 0.05). **(C)** Pathways enriched in significantly upregulated DEGs. **(D)** Representative immunofluorescence images of calreticulin staining (red) in LG acinar epithelial cells and corresponding quantification of fluorescence intensities. **(E)** Violin plot of *Pigr* expression in WT and TSP-1^-/-^acinar epithelial cells. **(F)** Levels of secretory IgA (sIgA) in tear samples of TSP-1^-/-^and WT mice. *q-value < 0.05, ***p-value < 0.001.

An analysis of the transcriptomic profiles of acinar epithelial cells in WT and TSP-1^-/-^LGs was performed to determine if gene expression patterns represented potential mechanisms underlying their functional loss and autoimmunity noted in TSP-1^-/-^mice. Overall, the expression pattern of mitochondrial ATPases, as illustrated in the heatmap in **Figure 2A**, was accompanied by significant DEGs (adjusted *p*< 0.05), including downregulated antioxidant enzyme genes like *Gpx7*and *Prdx4*and upregulated genes associated with ER stress—*Erp29*, *Ern1*, *Eif2ak3*, and *Atf6b*(**Figure 2B**). This coordinated shift in ATPases, antioxidant enzymes, and ER-stress-related transcripts in TSP-1^-/-^cells was also supported by the enrichment of pathways associated with ER stress among significantly altered DEGs (**Figure 2C**). Furthermore, in TSP-1^-/-^acinar cells, we detected significant downregulation of *Calm3*gene (**Figure 2B**) that encodes epithelial-specific calcium-sensing protein like calmodulin-like protein 3 (Calml3) and *Camk2b*gene that encodes the beta chain of calcium/calmodulin-dependent protein kinase II (CaMKII). Both Calml3 and CaMKII are known to play a protective role against oxidative stress-induced damage in epithelial cells (31, 32). These results altogether not only are consistent with the crucial role of mitochondrial ATPases in the high energy demands of acinar epithelial secretory processes (33) but also suggest the likely dysregulation of ROS-mediated oxidative stress resulting from increased mitochondrial ATPase activity. Resulting ER stress can contribute to functional loss of acinar epithelial cells.

Unresolved ER stress is known to activate NF-κB through several mechanisms and leads to inflammatory responses (34). This close interaction between two pathways is also reflected in the significantly increased expression of the gene encoding NF-κB protein (*Nfkb2*) (**Figure 2B**) in TSP-1^-/-^acinar epithelial cells consistent with previously reported formation of inflammasomes in these cells and the associated increased levels of inflammatory cytokine IL-1β (35). One of the downstream effects of ER stress is the transcription factor ATF6-driven production of ER chaperones, including calreticulin, as part of unfolded protein response (UPR) to enhance the protein folding capacity and mitigate the accumulation of misfolded proteins (36, 37). In TSP-1^-/-^acinar epithelial cells, the upregulation of both *Atf6*and calreticulin encoding *Calr*gene was detected (**Figure 2B**). This change was also confirmed by the increased detection of calreticulin protein by immunostaining (**Figure 2D**).

Additionally, in TSP-1^-/-^acinar epithelial cells, we detected a significant downregulation of *Aqp5*gene (**Figure 2A**), which encodes water channel aquaporin 5 (AQP5) along with a significant downregulation of tight junction proteins like claudins (*Cldn3*, *Cldn4*, *Cldn7*, *Cldn8*, and *Cldn34c2*) that are associated with the controlled movement of water that is essential for fluid secretion (38–40). These changes reflect disruption of vital components of water transport across the membrane and tear secretion and are consistent with dysregulated AQP5 reported in LG of Sjögren’s patients (41). Furthermore, in TSP-1^-/-^acinar epithelial cells, we noted a significant downregulation of the *Pigr*gene (**Figure 2E**) that encodes the polymeric immunoglobulin receptor (pIgR) required for the transcytosis of dimeric IgA produced by glandular plasma cells across acinar epithelial cells for delivery as sIgA into tear fluid (42). This finding is further supported by significantly reduced levels of sIgA in tears of TSP-1^-/-^mice (**Figure 2F**) as observed in Sjögren’s disease patients (43). Collectively, these results identify potential molecular mechanisms that contribute to the disrupted secretory function of acinar epithelial cells in TSP-1^-/-^LGs and the development of autoimmune inflammatory response.

### 3.3 Disruption of ion transport and calcium signaling contributes to secretory dysfunction of TSP-1-deficient duct epithelium

**Figure 3:**
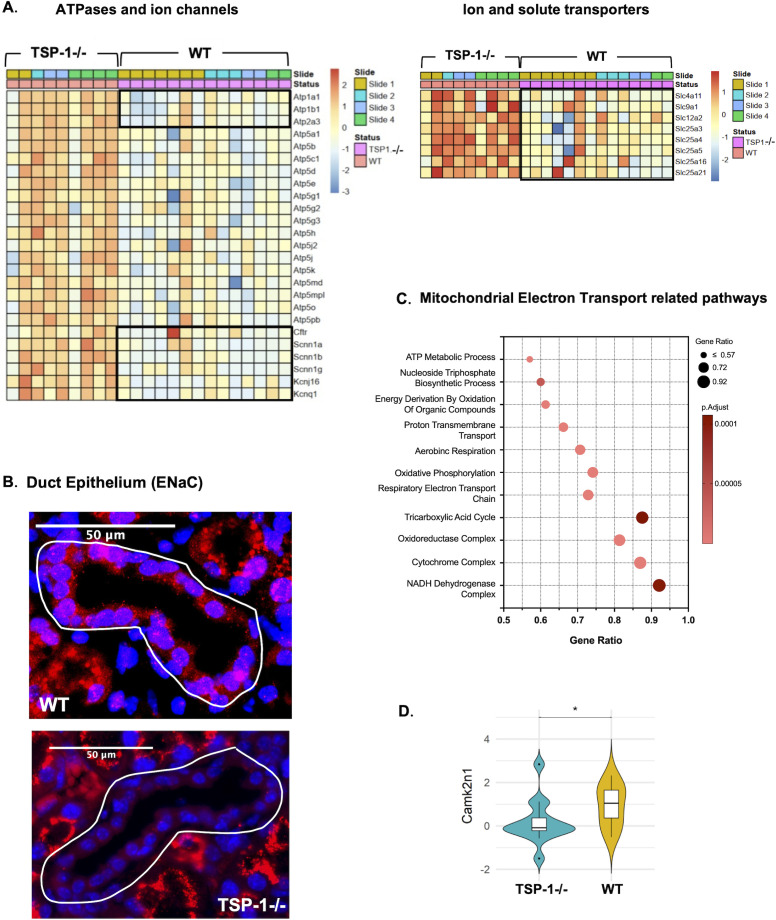
Ion transport and calcium signaling are altered in TSP-1-deficient duct epithelial cells. **(A)** Heatmaps illustrating the expression patterns of ATPases and ion channels (left) and ion and solute transport related genes (right) in duct epithelial cells. In the heatmap on the left side black boxes include Na^+^/K^+^and Ca^+^ATPases (top) and Cl-, Na+ and K+ ion channels (bottom) and mitochondrial ATPases outside boxes. **(B)** Representative immunofluorescence images of ENaC (epithelial sodium channel) (red) and nuclei (blue) in duct epithelium. White outlines highlight ductal structures. **(C)** Pathways enriched in significantly downregulated DEGs. **(D)** Violin plot comparing *Camk2n1 *expression in duct epithelial cells (*q-value <0.05).

Duct epithelial cells of the LG participate in tear secretion by fine tuning primary protein-rich lacrimal fluid produced by acinar epithelial cells (6, 44). This modification involves the addition and regulation of water and electrolytes like potassium (K+) and chloride (Cl-) ions, to adjust the composition, osmolarity and volume of final tear fluid. Some of the ion transporters used to achieve this adjustment include Na+/K+ ATP-ase (NKA), Na+/K+/Cl- co-transporter type 1 (NKCC1), cystic fibrosis transmembrane conductance regulator (CFTR) and Epithelial Na+ channel (ENaC). Genes encoding these transporters are *Atp1a1, Atp1b1*(NKAa1 and b1 subunits), *Slc12a2*(NKCC1), *Cftr*(CFTR) and *Scnn1a*, *Scnn1b*and *Scnn1g*(ENaC α, β and γ subunits). Additionally, many other transporters are involved in tear secretion. In our study, we detected significant downregulation of NKA, NKCC1, CFTR, ENaC encoding genes, potassium channels encoded by genes *Kcnj16, Kcnq1*, Sodium bicarbonate transporter *Slc4a11*, Sodium hydrogen exchanger *Slc9a1*and mitochondrial solute carriers that transport ADP/ATP *Slc25a4, Slc25a5, Slc25a16, Slc25a21* (**Figure 3A**). Significantly reduced expression of ENaC was confirmed by immunostaining as shown in **Figure 3B**. Additionally, we detected downregulated expression of mitochondrial ATPases in TSP-1^-/-^duct epithelial cells (**Figure 3A**). The reduced expression of mitochondrial ATPases most notably results from mitochondrial dysfunction and impaired electron transport chain which limits ATP production. This observed gene profile was also supported by a significant downregulation of genes associated with mitochondrial-electron-transport-related pathways (**Figure 3C**). Additionally, a significant downregulation of *Camk2n1*that encodes calcium/calmodulin dependent protein kinase II inhibitor was noted in TSP-1^-/-^duct epithelial cells (**Figure 3D**). These results together spatially correlate with a significant loss of secretory function of TSP-1^-/-^duct epithelial cells that likely contribute to compromised tear composition and correlate with ocular surface disease reported in these mice.

### 3.4 Impaired interactions of TSP-1-deficient myoepithelial cells with acinar epithelial cells underlies structural and functional damage of lacrimal gland

**Figure 4:**
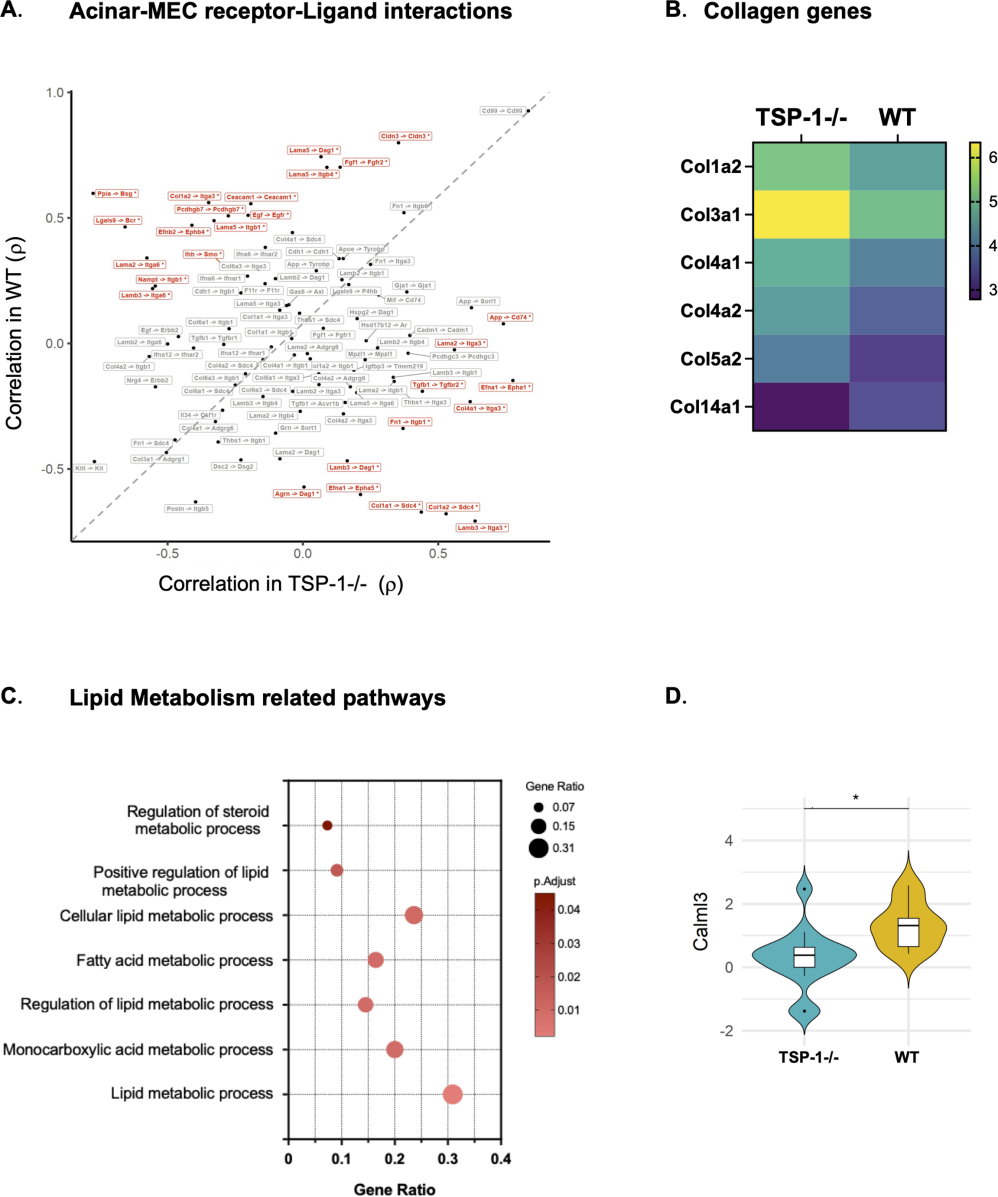
Changes in cellular interactions between MECs and acinar epithelial cells and in MEC contractile function reflecting structural and functional dysfunction in TSP-1-deficient glands. **(A)** Spatially correlated receptor–ligand pairs across acinar: MEC. Spearman’s rank correlation coefficients are plotted, and significant interactions are shown in red. **(B)** Heatmap showing the various collagen DEGs in MECs (adj *p*< 0.05). **(C)** Pathways enriched in downregulated DEGs in MECs. **(D)** Violin plot comparing *Calm3* expression in MECs (*q-value < 0.05).

In addition to the contractile capacity of MECs that is critical for the propulsion of tear fluid, their regenerative potential (45) is believed to play a significant role in the maintenance of the glandular structure (46, 47). Spatial profiling data offered us a unique opportunity to interrogate interactions among MECs and acinar epithelial cells in WT and TSP-1^-/-^LGs. We identified spatially defined receptor–ligand pairs co-expressed across ROIs in WT and TSP-1^-/-^glands. As shown in **Figure 4A**, although some receptor–ligand pairs were well correlated in both glands, many pairs were differentially correlated between WT and TSP-1^-/-^tissues. In normal WT LGs, highly correlated significant interactions included those between MEC-derived laminin-1 and integrin receptors on acinar epithelial cells (e.g., Lamb3 -> Itga6, Lama2 -> Itga6, Lama5 ->Itgb1, Lama5 ->Itgb4, Lama5 -> Dag1) known to promote acinar cell polarity, survival, and differentiation as well as interactions between adhesion molecules (Ceacam1 -> Ceacam1, Pcdhgb7 -> Pcdhgb7, Cldn3 -> Cldn3) important for the structural and functional integrity of LGs (48). Significant correlations between growth factors and receptors like Egf ->Egfr and Fgf -> Fgfr2 in WT LGs highlight EGFR signaling important in the secretory function of acinar epithelial cells and the role of growth factor FGF in promoting the proliferation and migration of MECs (49, 50). Lack of some or relatively fewer such interactions among significantly correlated receptor–ligand pairs in TSP-1^-/-^tissue coincides with the structural and functional loss observed in TSP-1^-/-^glands (7). The lack of significant Egf -> Egfr and Cldn3 -> Cldn3 correlations between TSP-1^-/-^MECs and acinar epithelial cells is supported by a significantly reduced expression of *Egf*in TSP-1^-/-^MECs (1.7-fold, adj. p<0.05) and that of *Cldn3*in TSP-1^-/-^acinar epithelial cell (1.5-fold, adj. *p*< 0.05). Although we did not detect a significantly reduced expression of laminin-1 in TSP-1^-/-^MECs, a significantly increased expression of laminin-degrading metalloproteinase, *Mmp2*, was detected in TSP-1^-/-^acinar epithelial cells (1.7-fold, adj. *p*< 0.05) that correlates with the pattern of receptor–ligand interactions.

Unlike laminin-1 in WT glands, in TSP-1^-/-^glands, highly correlated ECM proteins bound by integrins predominantly included fibronectin and collagens that are associated with epithelial repair following injury (51–53). In fact, significantly increased receptor–ligand correlations (e.g., Tgfb1 -> Tgfbr2, Fn1 ->Itgb1, Col4a1 ->Itga3, Col1a2 -> Sdc4) in TSP-1^-/-^gland suggestive of profibrotic process is accompanied by an increased expression of collagens (**Figure 4B**) known to play distinct roles in ECM remodeling during tissue repair (54). A similar increased expression of collagens was also reported in TSP^-/-^MECs previously in a microarray study (55). However, the expression of type XIV collagen, *Col14a1*, known to regulate the assembly and organization of collagen fibrils (56, 57) required to create the ECM scaffold, was significantly reduced, indicating impaired ECM organization. Furthermore, a significant correlation between amyloid precursor protein and MHC invariant chain (App -> Cd74) is consistent with an increased CD74 expression in TSP-1^-/-^acinar epithelial cells (1.7-fold, adj. *p*< 0.05). A similar increased epithelial CD74 expression is reported in diverse tissue injury diseases and inflammatory responses including autoimmune diseases (58–60).

We also detected enrichment of pathways associated with lipid metabolism among differentially downregulated genes in TSP-1^-/-^MECs (**Figure 4C**). As lipid metabolism is known to support smooth muscle contraction through various mechanisms (61, 62), this finding suggests a potential loss of contractile responses in TSP-1^-/-^MECs. This possibility is also supported by a significantly reduced expression of calcium-sensor-encoding gene *Calml3*(**Figure 4D**), further corroborating previously reported *in vitro*observations of altered calcium signaling and contractile responses in primary cultures of TSP-1^-/-^MECs (55). Collectively, our findings indicate that the disruption of distinct interactions of MECs with acinar epithelial cells and their contractile function significantly contributes to the structural and functional loss observed in TSP-1^-/-^LGs.

### 3.5 Periductal immune infiltrates in TSP-1-deficient lacrimal glands form GCs

**Figure 5:**
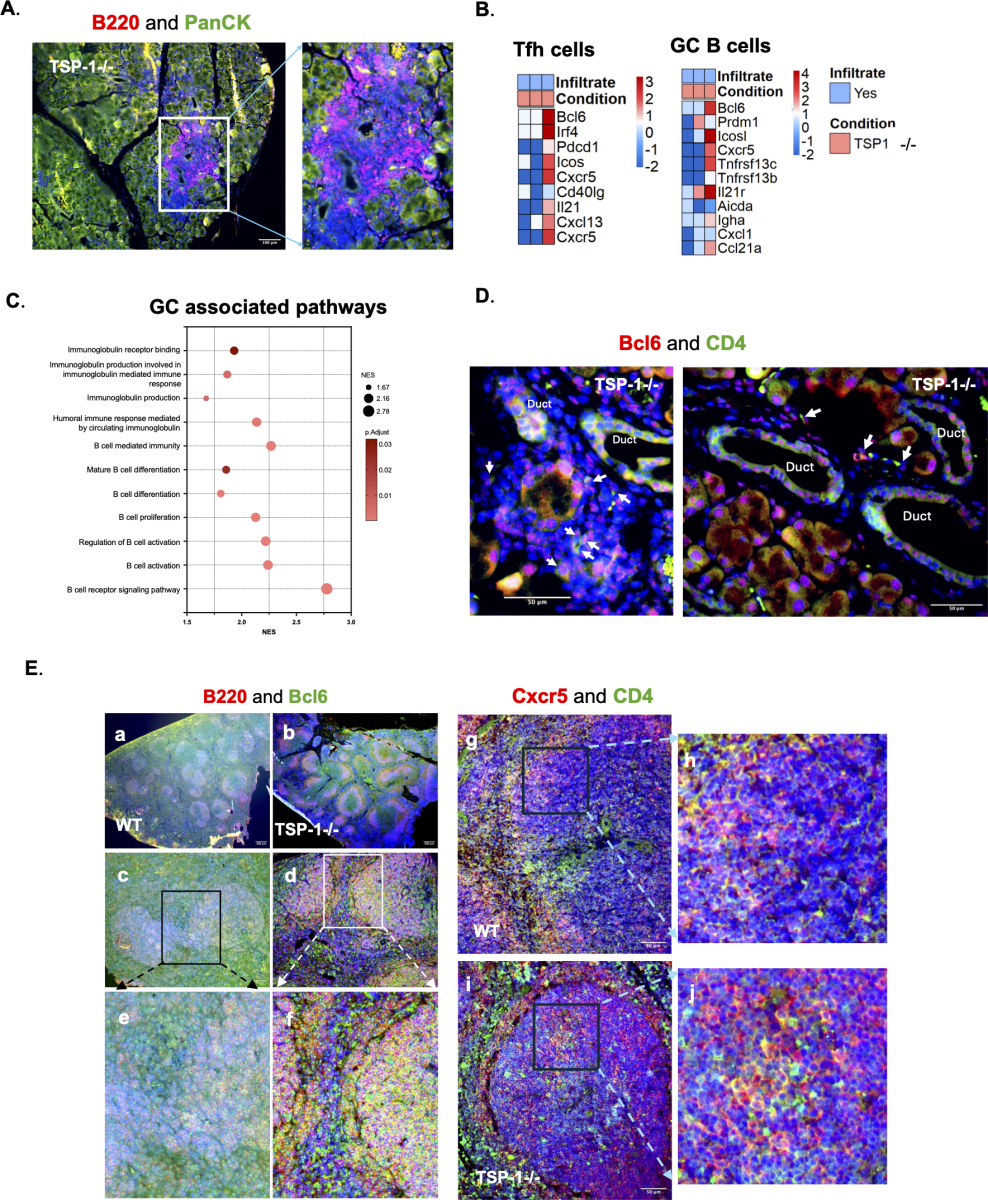
Spontaneous germinal centers containing B cells and Tfh cells in the lacrimal gland and spleen of TSP-1- deficient mice. **(A)** Immunofluorescence staining for B220 and PanCK showing the predominance of B cells in periductal infiltrates detected in TSP-1^-/-^LG. **(B)** Heatmaps of gene expression profiles of GC B and Tfh cells in three different areas containing periductal infiltrates in TSP-1^-/-^LG. **(C)** Pathways enriched in significantly upregulated DEGs in periductal immune cells. **(D)** Representative immunofluorescence images showing Bcl6+ staining and Bcl6+CD4+(Tfh) cells in the periductal infiltrates (white arrows). **(E)** Immunofluorescent images of spleen sections stained for GC B (Bcl6+B220+) cells (subpanels a–f: the boxed regions in c and d are enlarged in e and f, respectively, below to show GCs in the follicle and Tfh cells showing only Bcl6 staining at the periphery and within the follicle) and Tfh (CXCR5+ CD4+) cells (subpanels g & i: the boxed regions in g and i are enlarged in h and j, respectively, on the right to show Tfh cells in the follicle). Co-expression of markers is indicated by the yellow color.

As reported previously, we observed periductal and perivascular lymphoid aggregates characteristic of SjD pathology in TSP-1^-/-^, but not WT LGs. In our DSP study, CD45 staining in ROIs containing immune infiltrates was very weak, which we confirmed by staining additional slides (**Supplementary Figure 3**). Among immune cells, low CD45 expression is reported in B cell subsets, which is related to their enhanced survival and autoimmunity (63–65). Staining of these aggregates with B cell marker (B220) confirmed B cell predominance (**Figure 5A**). Expression of markers associated with germinal center B cells (GC B) and T follicular helper (Tfh) cells was detectable in the transcriptomic profile of three ROIs containing such aggregates in TSP-1-/- LGs (**Figure 5B**), suggesting the presence of activated B cells. Moreover, a pathway analysis of DEGs in the aggregate revealed significant enrichment of pathways associated with the formation of the germinal center (**Figure 5C**), suggesting lymphoid aggregates to be active germinal centers (GCs). We also detected CD4-positive cells that stained for Bcl6, the lineage-defining transcription factor of Tfh cells. These cells were found in GC as well as among periductal immune cells in TSP-1^-/-^LGs (**Figure 5D**), further validating our gene expression profile of lymphoid aggregates. To determine if the observed GCs in TSP-1^-/-^LGs correlate with spontaneous splenic GC formation that is commonly associated with systemic autoimmunity, we evaluated WT and TSP-1^-/-^spleen sections. Relative to WT spleen, more activated follicles marked by the presence of B220+Bcl6+ (yellow) GC B cells and B220-Bcl6+ (green) Tfh cells are seen in TSP-1^-/-^spleen (**Figures 5E, a–f**). We further confirmed the Tfh cells as CXCR5+CD4+ (yellow) cells as seen in **Figures 5E, g–j**, near the periphery and within the follicle in TSP-1^-/-^spleens, in comparison to WT spleen. These results together support the presence of ectopic GCs in LGs that correlates with spontaneous splenic GCs in TSP-1^-/-^mice consistent with their systemic autoimmunity.

### 3.6 Immune cells adjacent to duct epithelial cells in TSP-1-deficient lacrimal gland include antigen-presenting cells capable of recruiting Tfh and GC B cells

**Figure 6:**
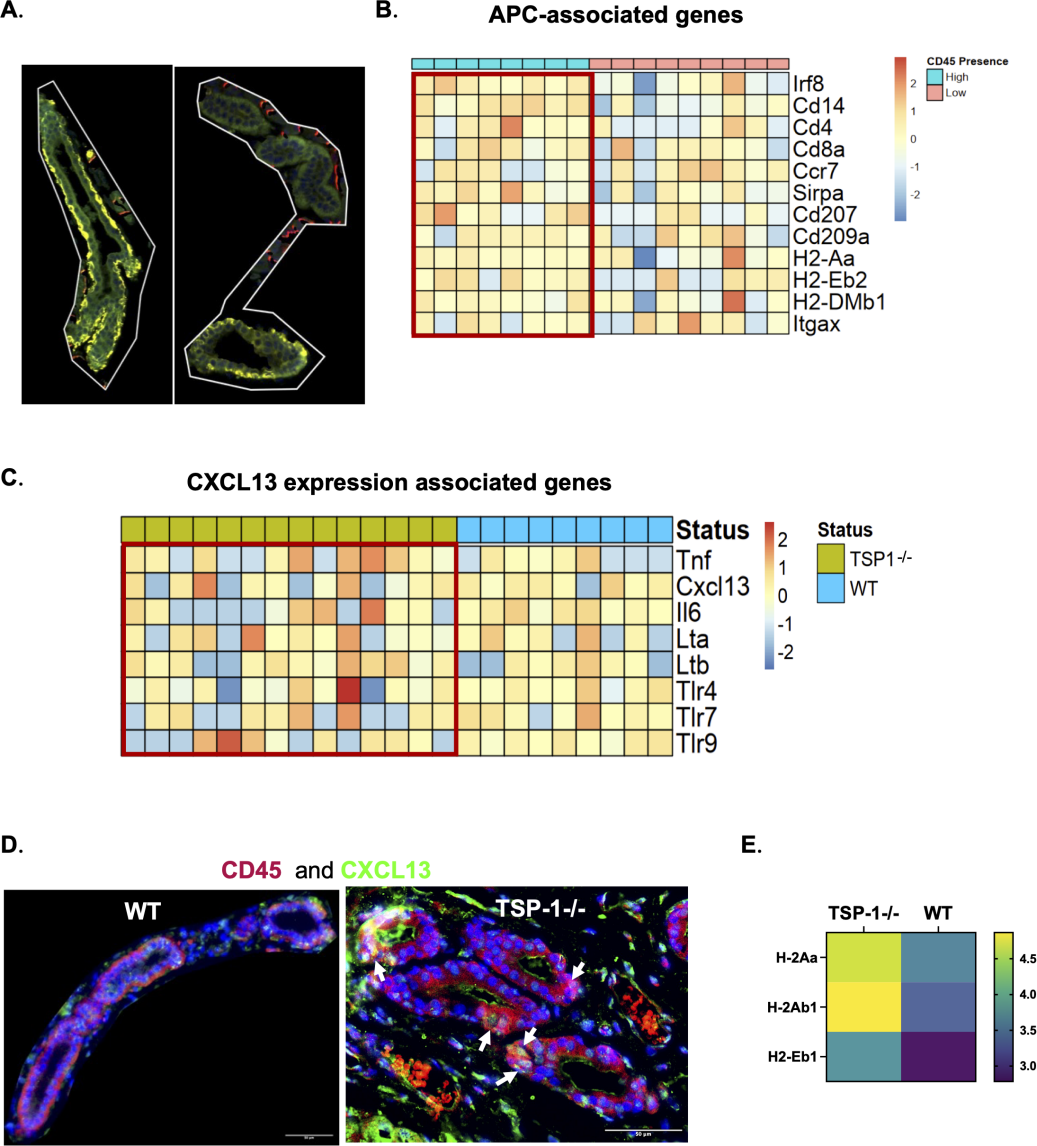
Spatial relationship of duct epithelial cells with antigen-presenting cells and their pro-inflammatory gene signatures in TSP1-deficient lacrimal glands. **(A)** Representative images showing ROIs containing PanCK (green) positive duct epithelial cells and CD45 (yellow) positive immune cells with variable (high and low) staining intensities. **(B)** Heatmap of APC-associated gene expression mapped to ROIs containing ducts with high (red box) vs. low CD45 staining intensity correlates with higher CD45 staining. **(C)** Heatmap of the upregulated pattern of CXCL13 and related inflammatory mediators in APCs among ductal ROIs from TSP-1^-/-^(red box) relative to WT LGs. **(D)** Representative images of WT and TSP-1^-/-^ducts showing CXCL13 co-localization with CD45 (white arrows) in the latter. **(E)** Heatmap showing the significantly altered expression of MHC class II genes in duct epithelial cells (adj *p*< 0.05).

In our DSP analysis, ROIs containing ducts included CD45 staining in close proximity to PanCK- stained duct epithelial cells in both WT and TSP-1^-/-^LGs as shown in **Figure 6A**. Some duct ROIs exhibited higher CD45 staining intensity than others, with a greater proportion displaying elevated CD45 staining in TSP-1^-/-^glands (40%) compared to WT glands (22%). We compared the transcriptional profiles of ductal ROIs with high and low CD45 staining intensity and observed that the expression of genes associated with antigen-presenting cells (APCs) correlated with the CD45 intensity (**Figure 6B**). This correlation suggested the presence of APCs among immune cells occupying periductal spaces, indicating a previously unrecognized spatial relationship between ductal epithelial cells and APCs in LGs.

Prior studies have identified APCs—particularly monocytes, macrophages and dendritic cells—as sources of CXCL13 under inflammatory conditions through the activation of TLR7/9, TLR4, and cytokine signaling pathways such as TNF-α (66, 67). Considering the periductal localization of infiltrating CXCR5+ Tfh cells and GC B cells in TSP-1^-/-^LGs, we compared the expression of CXCL13 and related inflammatory mediators in immune cells adjacent to the ductal epithelium between TSP-1^-/-^and WT tissues. As shown in **Figure 6C**, several ROIs from TSP-1^-/-^glands displayed an upregulated expression of these genes, and immunostaining confirmed CXCL13 co-localization with CD45 (**Figure 6D**), supporting the formation of periductal GCs. Furthermore, ductal epithelial cells in TSP-1^-/-^glands exhibited a significantly increased MHC class II expression relative to WT LGs (**Figure 6E**), suggesting their potential involvement in autoantigen presentation during glandular inflammation.

### 3.7 Duct epithelial cells in TSP-1-deficient lacrimal glands provide the microenvironment to generate GCs

**Figure 7:**
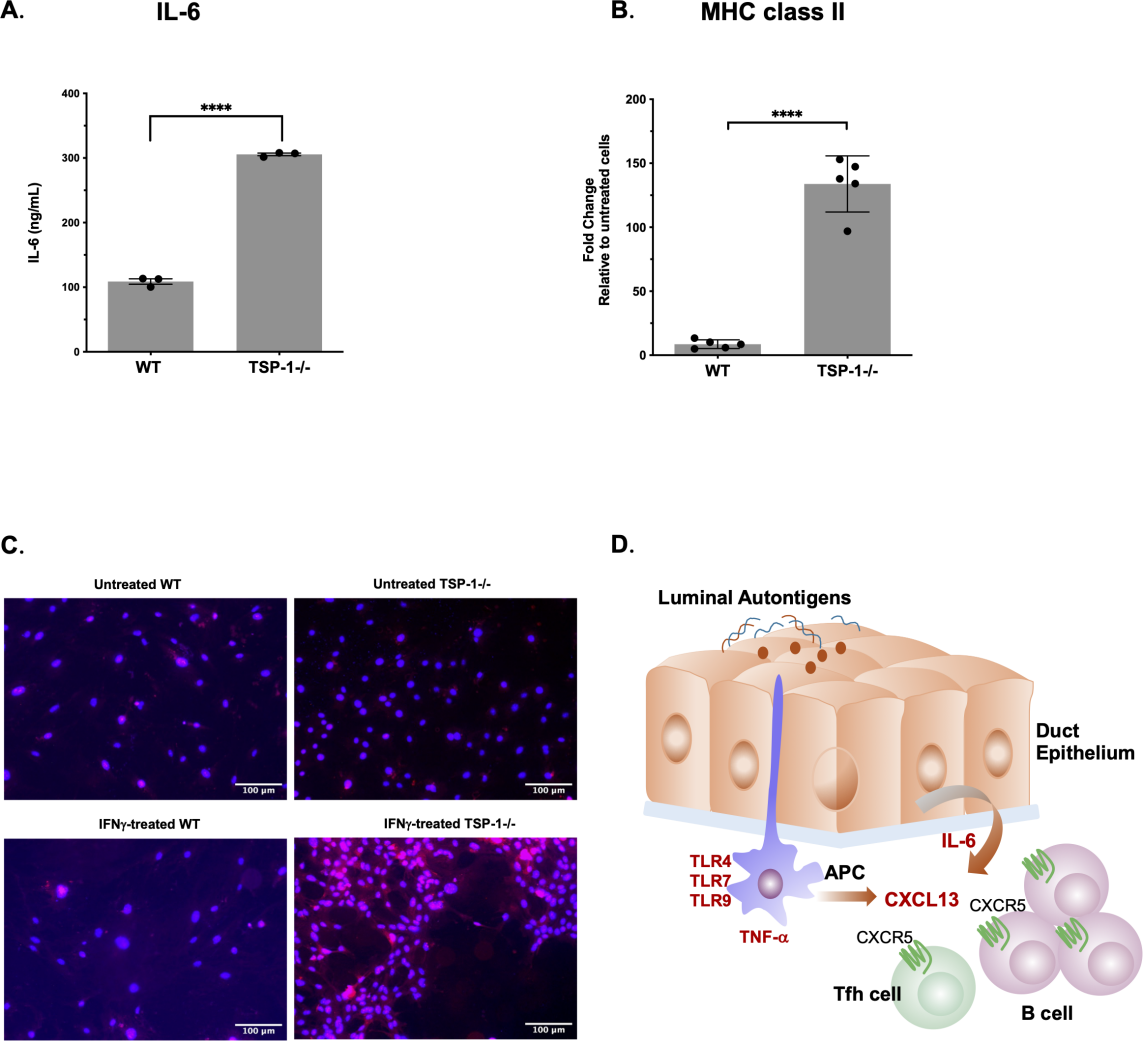
Duct epithelial cells from TSP-1-deficient lacrimal gland secrete increased IL-6 and express higher levels of MHC class II under inflammatory conditions. **(A)** Levels of IL-6 detected in 24-h supernatants of primary cultures of duct epithelial cells. **(B)** Expression of MHC class II gene in IFN-γ-stimulated primary duct epithelial cell cultures. Data presented as mean ± SEM, ****p< 0.001. **(C)** Immunostaining of MHC class II in untreated and IFN-γ-stimulated primary WT (left panels) and TSP-1-/- (right panels) duct epithelial cell cultures. **(D)** Schematic representation of molecular mechanisms underlying the development of periductal immune infiltrates and spontaneous GC formation. Capturing luminal autoantigens by APCs adjacent to duct epithelial cells can induce their expression of pro-inflammatory molecules TNF-α and CXCL13 to help recruit CXCR5-expressing Tfh and GC B cells. Duct-epithelial-cell-derived IL-6 can provide a microenvironment permissive for the proliferation of recruited cells and facilitate the formation of spontaneous periductal GC.

The proximity of duct epithelial cells to APCs suggested a possibility of duct-epithelial-cell-derived factors shaping the local immune response induced by APCs. In autoimmune pathology, IL-6 is a key cytokine known to drive the polyclonal activation of B cells to facilitate the emergence of autoreactive clones, promote the differentiation of Tfh cells that help in GC formation, and act synergistically with CXCL13 to recruit and retain B and Tfh cells (68, 69). We detected several duct epithelial ROIs in TSP-1^-/-^LGs with a relatively higher expression of IL-6. To validate this finding and determine if duct epithelial cells contribute to microenvironment supportive of periductal GC formation, we generated primary cultures of LG-derived duct epithelial cells as described in “Materials and methods”. These cultures were validated by confirming their expression of epithelial cell (PanCK) and duct epithelial cell (CFTR) markers (70) and the absence of myoepithelial and acinar cells as evident from minimal to no detectable immunostaining for α-SMA and Rab3d, respectively (**Supplementary Figure 4**). Duct epithelial cell cultures derived from WT and TSP-1^-/-^LGs were used to collect 24-h culture supernatants to compare their secretion of IL-6. As shown in **Figure 7A**, significantly increased IL-6 levels were detectable in TSP-1^-/-^duct epithelial cell cultures as compared to WT control cultures. Another way epithelial cells are known to influence the local immune response is by functioning as non-professional APCs by expressing MHC class II molecules in response to IFN-γ and participate in activation of effector CD4+ T cells (71). We next assessed if TSP-1^-/-^duct epithelial cells differed from WT controls in their ability to respond to IFN-γ by determining their expression of MHC class II by RT-PCR after exposure to IFN-γ as described in methods. As shown in **Figure 7B**, a significantly increased expression of MHC class II was detectable in TSP-1^-/-^duct epithelial cells as compared to the WT controls. This result was confirmed by immunostaining of MHC class II in these cells (**Figure 7C**). Collectively, these results support the potential of duct epithelial cells to shape local immune response by facilitating the formation of periductal GCs in TSP-1^-/-^LGs.

## 4 Discussion

Previous studies have reported that TSP-1 mediates TGF-β activation, immune tolerance, and maintenance of LG function, and its loss underlies autoimmunity associated with SjD (9, 17, 18). Deletion of TSP-1 in mice produces a model that closely mimics the gradual disease progression observed in SjD patients, providing a robust system to dissect the cellular and molecular mechanisms driving pathology. Using DSP of LG tissues from healthy and TSP-1 deficient mice, we identified spatial interactions between epithelial cells and immune cells contributing to autoimmunity and glandular damage. This approach complemented scRNA-seq findings by adding spatial context, revealing potential epithelium-driven molecular mechanisms underlying LG structural and functional loss. Moreover, our results highlight tissue microenvironment changes that promote periductal infiltrates and germinal center (GC) formation, hallmarks of SjD pathology.

Endoplasmic reticulum (ER) stress has emerged as a key factor in autoimmunity, including rheumatic diseases (72). The unfolded protein response (UPR), activated by ER stress, regulates both innate and adaptive immunity, yet its activation in SjD-affected LG was previously unclear. Transcription profiling of acinar epithelial cells revealed upregulated ER stress pathway in TSP-1^-/-^LGs, mirroring findings in salivary glands of SjD patients (73) and in models lacking ER-resident chaperone protein (74). Furthermore, ER stress can trigger pro-inflammatory NF-kB signaling, NLRP3 inflammasome activation, and cell death (34, 75) consistent with previously observed inflammasome formation and apoptosis in TSP-1^-/-^LG epithelial cells (7, 35). Elevated calreticulin, a downstream ER stress marker, supports this phenotype (36). Its upregulation is linked to immunogenic cell death (ICD) and aberrant immune activation, contributing to autoantibody production (76), and is involved in the presentation of Ro60 (SSA) epitopes (77, 78). These mechanisms collectively explain the presence of anti-Ro and anti-calreticulin autoantibodies in TSP-1^-/-^mice and SjD patients (7, 79). Furthermore, the structural LG damage in SjD also manifests as reduced *Pigr*expression, disrupting IgA transcytosis that lowers tear sIgA levels. Since sIgA modulates commensal microbiota in mucosal inflammatory disease (80, 81), the reduced tear sIgA correlates with previously reported microbial alterations in TSP-1^-/-^mice (82) and may serve as a biomarker of glandular epithelial damage.

Beyond acinar cell dysfunction, we identified mechanisms underlying duct epithelial impairment, which are crucial to regulate the tear composition which maintains the integrity of the ocular surface. These cells maintain ionic balance through transporter and channel expression, producing K^+^- and Cl^--^rich tears (44). In both rabbit SjD model and TSP-1^-/-^mice, a reduced expression of Na^+^/K^+^ATPases and ion channel expression correlates with LG dysfunction and ocular surface disease (83, 84). The downregulation of mitochondrial ATP synthases and electron transport chain components suggests mitochondrial dysfunction, implicated in autoimmune disease pathogenesis (85). Damaged mitochondria release nucleic acids and danger-associated molecular patterns (DAMPs), amplifying inflammation (86, 87). A reduced expression of calcium-signaling-related molecule (*Calml3*) in TSP-1 ^-/-^MECs and acinar cells further indicates impaired contractile and secretory function, respectively (7, 55, 88). Spatial analysis also revealed profibrotic MEC–acinar interaction and ECM remodeling signatures, consistent with fibrotic progression correlated with dry eye in patients and glandular atrophy observed in advanced SjD, suggesting their potential as early biomarkers of disease progression (89, 90).

Chronic LG inflammation in SjD features periductal and perivascular B- and T-cell infiltrates that form GCs supporting local autoantibody production (15, 91). The LG epithelial contribution that fosters GC formation has remained undefined. Our study demonstrates B-cell-dominated periductal infiltrates and spontaneous GC formation in TSP-1^-/-^LGs and spleen, consistent with serological SjD markers in mouse models as well as patients (7, 15, 92, 93). Spatial mapping identified APCs adjacent to duct epithelial cells—an observation not previously described—suggesting autoantigen sampling from lacrimal secretion (**Figure 7D**). The expression of signaling molecules TLRs and TNF-α known to induce Tfh and GC B cell-attracting chemokine, CXCL13, by some of these APCs explains the periductal location of infiltrates and GCs in LGs (66, 67). Additionally, we report for the first time the ability of duct epithelial cells in LG to express MHC class II consistent with IFN-γ-induced expression in mucosa, supporting their potential role in the immune surveillance and shaping of the local immune response (71, 94). *In vitro*, TSP-1^-/-^duct epithelial cells showed similar IFN-γ responsiveness and increased IL-6 production, which can sustain GC reactions by promoting Tfh and B-cell differentiation (69, 95). These findings collectively delineate spatial and molecular interactions that correlate with the development of periductal immune infiltrates in SjD-associated LG pathology.

In summary, our results help build on prior studies by dissecting cellular ecosystem in LGs under both normal and disease condition. Spatial transcriptomic profiling of LGs revealed epithelial cell subtypes and spatial signatures underlying their functional loss, active participation in promoting and sustaining autoimmune inflammation (beyond serving as passive targets), GC-promoting factors, potential early fibrotic markers, and potential tear biomarker indicative of glandular damage. While the low replicate number in this exploratory study limits the confirmatory power, these results have enabled candidate prioritization from high-plex spatial data for further validation using conditional transgenic lines or *in vitro* co-culture systems to establish mechanistic causality.

## Data Availability

The datasets presented in this study can be found in online repositories. The names of the repository/repositories and accession number(s) can be found below: https://www.ncbi.nlm.nih.gov/geo/, GSE312237.

## Supplementary material

Supplementary Figure 1 Digital spatial profiling with whole transcriptome atlas (WTA DSP) of mouse lacrimal glands. Immunofluorescence images of FFPE sections from WT and TSP-1^-/-^LGs stained with morphology markers with each section analyzed for the transcriptome of marked ROIs. The color legend indicates the target for each fluorophore-conjugated antibody used as morphology marker to identify cell types —epithelial cells (PanCK), immune cells (CD45), myoepithelial cells (α-SMA), and nuclear stain (SYTO 13).

Supplementary Figure 2 Epithelial cell clustering in the analyzed ROIs. Principal component analysis plots showing epithelial cell clusters detected in ROIs marked in WT and TSP-1-deficient LG sections.

Supplementary Figure 3 Weak CD45 staining of immune infiltrates in TSP-1-deficient lacrimal gland. **(A)**Lacrimal gland tissue section from TSP-1^-/-^mouse showing infiltrate containing ROI stained weakly (yellow) with morphology marker anti-CD45 (clone EM-05 and fluorescent secondary antibody) used in DSP analysis. Immune infiltrates are detectable with blue nuclear staining within marked ROI. **(B)**Confirmation of weak CD45 staining (yellow) pattern within immune infiltrates in TSP-1^-/-^LG section stained with fluorescence-conjugated anti-CD45 (clone 30-F11) (right), control antibody (left), and DAPI for nuclear staining.

Supplementary Figure 4 Primary cultures of lacrimal gland epithelial cells. **(A)**Adherent primary cultures stained predominantly with epithelial cell marker (PanCK) and duct epithelial marker (CFTR) with negligible (<5%) staining for myoepithelial marker (SMA). **(B)**Primary cultures immunostained with phalloidin (green) and Amplex Red reagent (to stain secretory vesicles containing peroxidase) or green-fluorochrome-conjugated anti-Rab3d (secretory vesicle marker); arrowheads indicate positively stained secretory vesicles of acinar epithelial cells (<5% of total cells). Nuclear stain DAPI (blue). Images at ×200 magnification.
