# Correction: Spatial transcriptomic profiling identifies lacrimal-gland-epithelial cell-driven mechanisms underlying autoimmunity in Sjögren’s disease

**DOI:** 10.3389/fimmu.2026.1832246

**Published:** 2026-04-06

**Authors:** Shivali Gupta, Athanasios Ploumakis, Nikolaos Kalavros, Sharmila Masli

**Affiliations:** 1 Department of Ophthalmology, Boston University Chobanian & Avedisian School of Medicine, Boston, MA, United States; 2 Spatial Technologies Unit, Beth Israel Deaconess Medical Center, Harvard Medical School Initiative for RNA Medicine, Boston, MA, United States

**Keywords:** acinar epithelial cells, antigen presenting cells, autoimmunity, duct epithelial cells, lacrimal gland, Sjögren’s disease, spatial transcriptomics

There was a mistake in **Figure 3**, panel A as published. Group labels included above the two heat maps “TSP-1-/-” and “WT” were inadvertently switched. The status indicated by the group of first 9 orange columns was labeled as “TSP-1-/-” and the group of following 14 pink columns was labeled as “WT”. The corrected **Figure 3**, panel A appears below.

**Figure 3:**
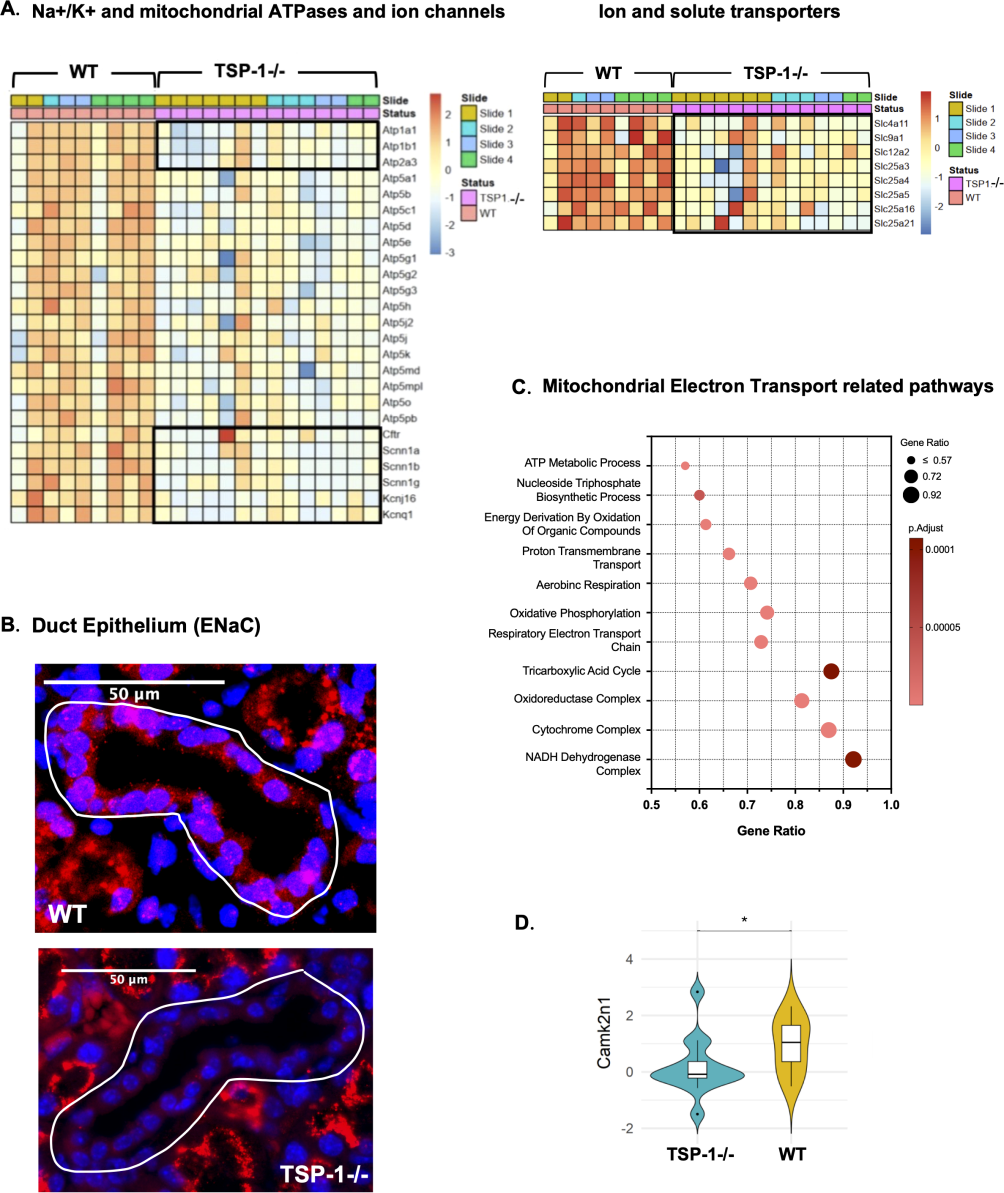
Ion transport and calcium signaling are altered in TSP-1-deficient duct epithelial cells. **(A)** Heatmaps illustrating the expression patterns of ATPasesand ion channels (left) and ion and solute transport related genes (right) in duct epithelial cells. In the heatmap on the left side black boxes includeNa + /K +and Ca + ATPases (top) and Cl-, Na+ and K+ ion channels (bottom) and mitochondrial ATPases outside boxes. **(B)** Representativeimmunofluorescence images of ENaC (epithelial sodium channel) (red) and nuclei (blue) in duct epithelium. White outlines highlight ductalstructures. **(C)** Pathways enriched in significantly downregulated DEGs. **(D)** Violin plot comparing Camk2n1 expression in duct epithelial cells (*q-value <0.05).

The original version of this article has been updated.

