# Supplementary figures and images for: Spatial transcriptomic profiling identifies lacrimal-gland-epithelial cell-driven mechanisms underlying autoimmunity in Sjögren’s disease
Source: Front Immunol. 2026 Mar 5;17:1759347. doi: 10.3389/fimmu.2026.1759347 (PMC12999407; doi:10.3389/fimmu.2026.1759347)

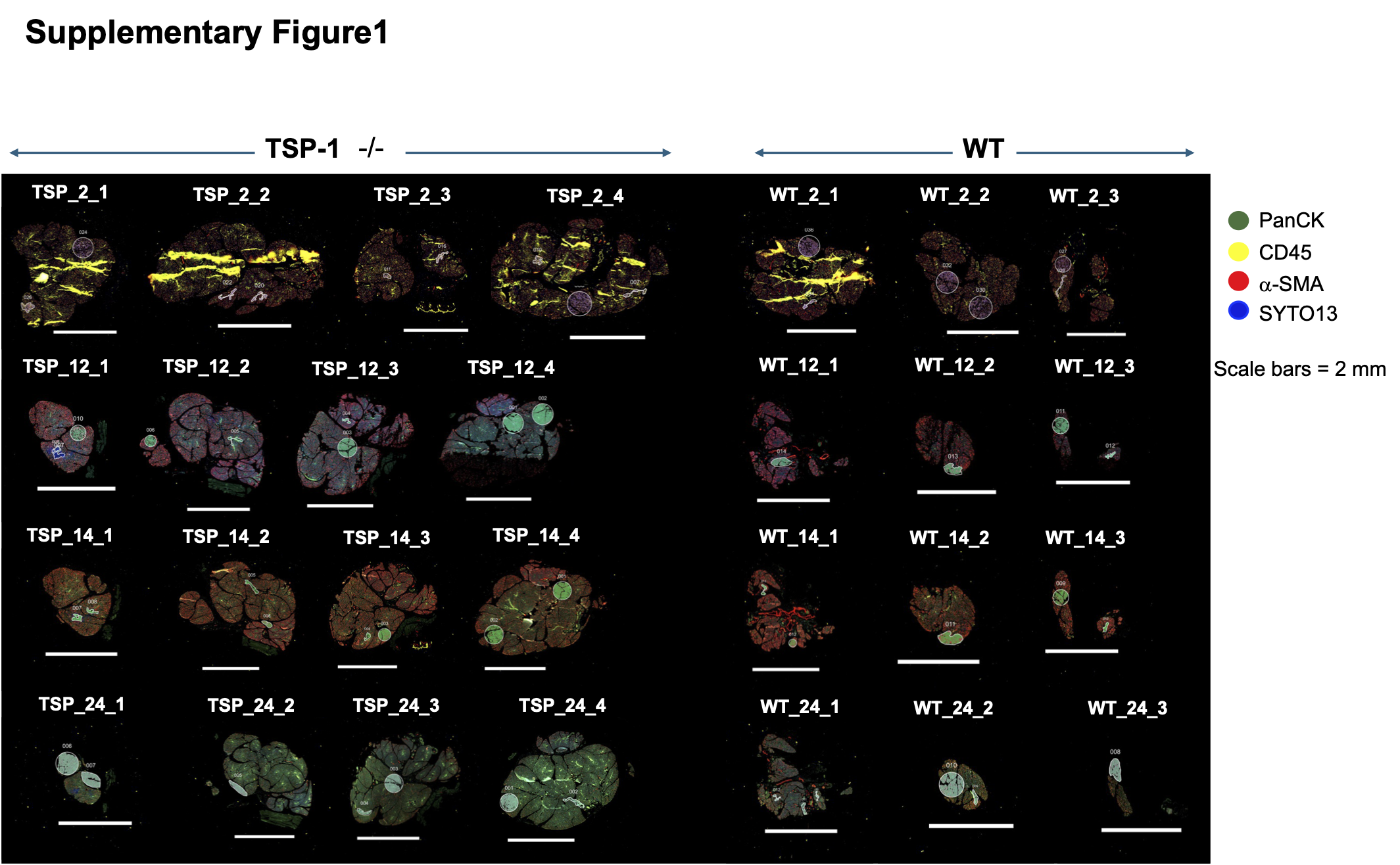

Supplement: Supplementary Figure 1 — Digital spatial profiling with whole transcriptome atlas (WTA DSP) of mouse lacrimal glands. Immunofluorescence images of FFPE sections from WT and TSP-1-/-LGs stained with morphology markers with each section analyzed for the transcriptome of marked ROIs. The color legend indicates the target for each fluorophore-conjugated antibody used as morphology marker to identify cell types —epithelial cells (PanCK), immune cells (CD45), myoepithelial cells (α-SMA), and nuclear stain (SYTO 13). [file Image1.tiff]

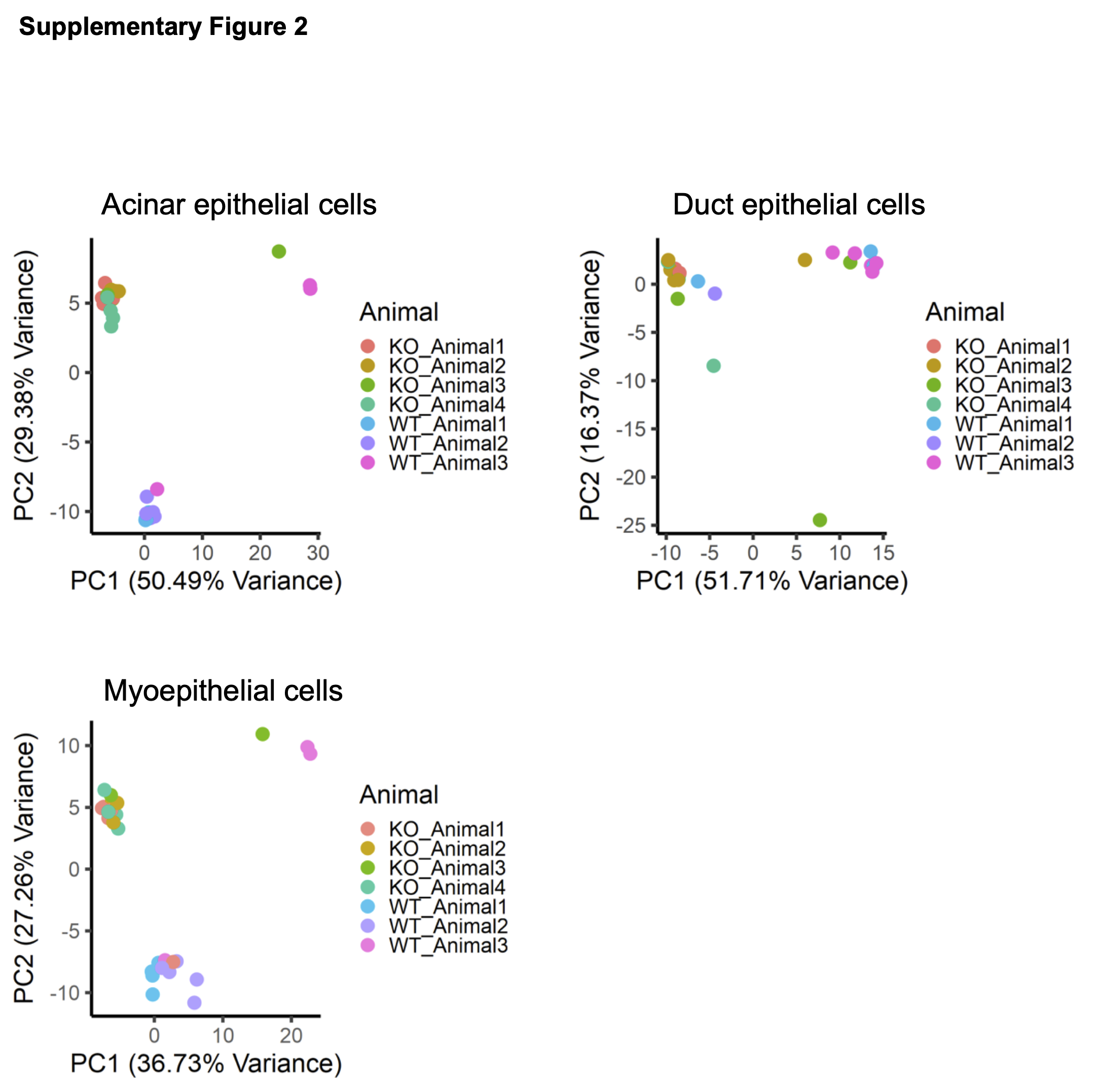

Supplement: Supplementary Figure 2 — Epithelial cell clustering in the analyzed ROIs. Principal component analysis plots showing epithelial cell clusters detected in ROIs marked in WT and TSP-1-deficient LG sections. [file Image2.tiff]

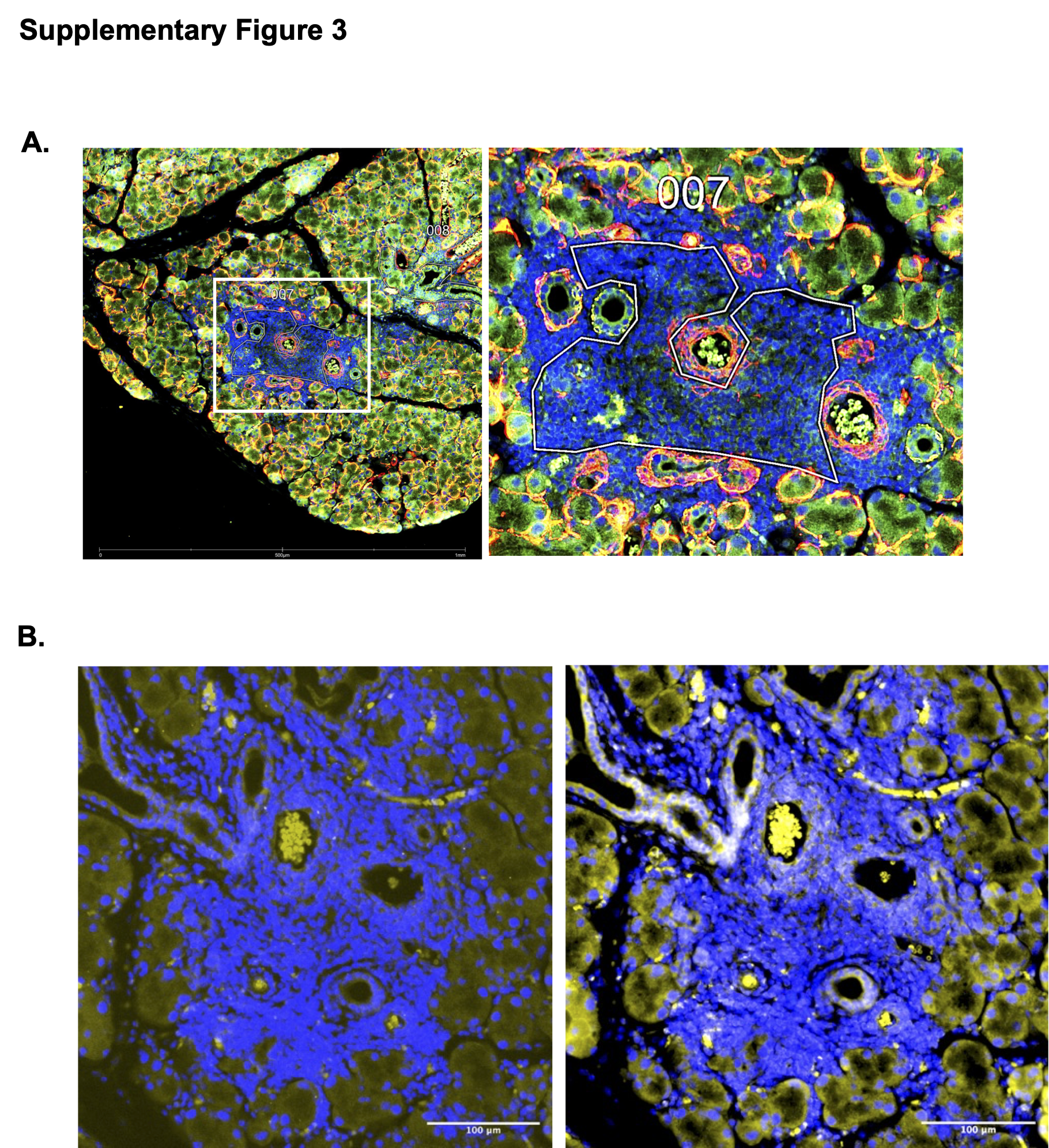

Supplement: Supplementary Figure 3 — Weak CD45 staining of immune infiltrates in TSP-1-deficient lacrimal gland. (A)Lacrimal gland tissue section from TSP-1-/-mouse showing infiltrate containing ROI stained weakly (yellow) with morphology marker anti-CD45 (clone EM-05 and fluorescent secondary antibody) used in DSP analysis. Immune infiltrates are detectable with blue nuclear staining within marked ROI. (B)Confirmation of weak CD45 staining (yellow) pattern within immune infiltrates in TSP-1-/-LG section stained with fluorescence-conjugated anti-CD45 (clone 30-F11) (right), control antibody (left), and DAPI for nuclear staining. [file Image3.tiff]

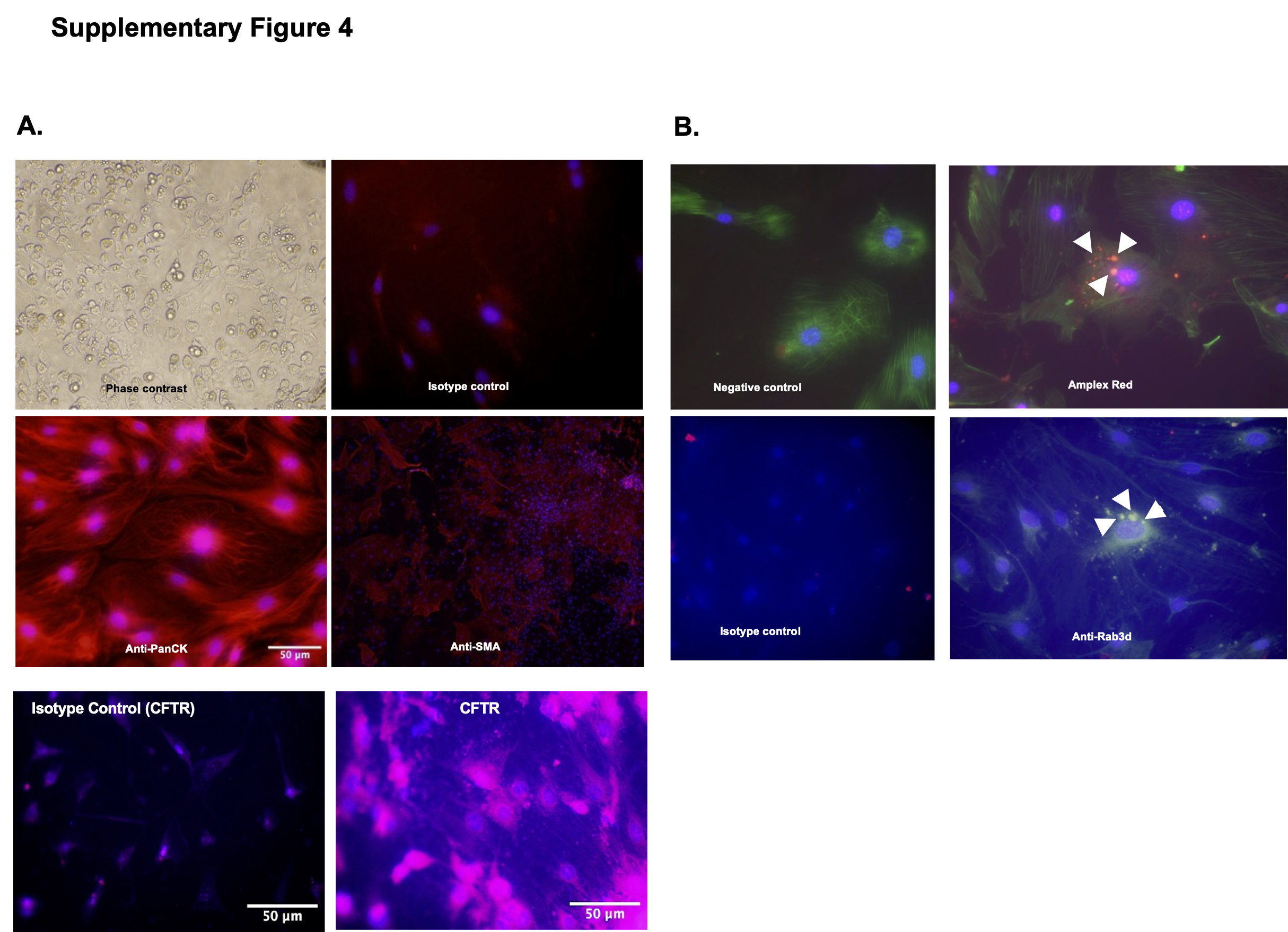

Supplement: Supplementary Figure 4 — Primary cultures of lacrimal gland epithelial cells. (A)Adherent primary cultures stained predominantly with epithelial cell marker (PanCK) and duct epithelial marker (CFTR) with negligible (<5%) staining for myoepithelial marker (SMA). (B)Primary cultures immunostained with phalloidin (green) and Amplex Red reagent (to stain secretory vesicles containing peroxidase) or green-fluorochrome-conjugated anti-Rab3d (secretory vesicle marker); arrowheads indicate positively stained secretory vesicles of acinar epithelial cells (<5% of total cells). Nuclear stain DAPI (blue). Images at ×200 magnification. [file Image4.tiff]

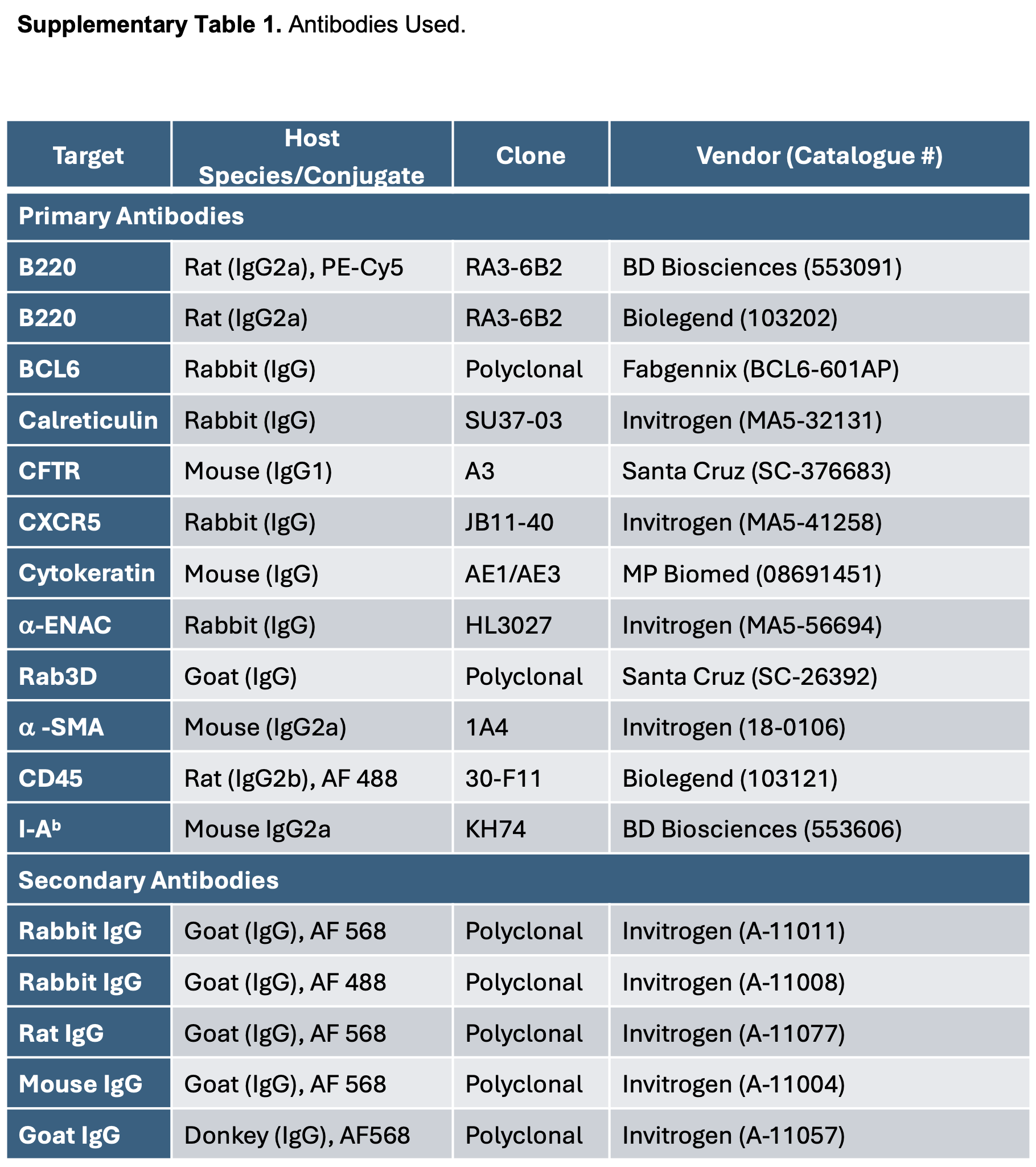

Supplement: Supplementary file 5 [file Supplementaryfile1.tiff]
